# Monitoring conservation effects on a Chinese indigenous chicken breed using major histocompatibility complex B-G gene and DNA Barcodes

**DOI:** 10.5713/ajas.17.0627

**Published:** 2018-04-11

**Authors:** Yunjie Tu, Jingting Shu, Gaige Ji, Ming Zhang, Jianmin Zou

**Affiliations:** 1 Poultry Institute, Chinese Academy of Agricultural Sciences, Yangzhou, Jiangsu, 225125, China; 2 Key lab of Poultry Genetics and Breeding in Jiangsu Province, Yangzhou, Jiangsu, 225125, China

**Keywords:** Chicken, Conservation, Major Histocompatibility Complex (*MHC*), DNA Barcodes, Genetic Diversity

## Abstract

**Objective:**

We report monitoring conservation effect for a Chinese indigenous chicken (Langshan) breed using major histocompatibility complex (*MHC*) and DNA barcords.

**Methods:**

The full length of *MHC B-G* gene and mitochondrial cytochrome oxidase I (*COI*) gene in generations 0, 5, 10, 15, 16, and 17 was measured using re-sequencing and sequencing procedures, respectively.

**Results:**

There were 292 single nucleotide polymorphisms of *MHC B-G* gene identified in six generations. Heterozygosity (He) and polymorphic information content (PIC) of *MHC B-G* gene in generations 10, 15, 16, and 17 remained stable. He and PIC of *MHC B-G* gene were different in six generations, with G10, G15, G16, G17 >G5>G0 (p<0.05). For the *COI* gene, there were five haplotypes in generations 0, 5, 10, 15, 16, and 17. Where Hap2 and Hap4 were the shared haplotypes, 164 individuals shared Hap2 haplotypes, while Hap1 and Hap3 were the shared haplotypes in generations 0 and 5 and Hap5 was a shared haplotype in generations 10, 15, 16, and 17. The sequence of *COI* gene in 6 generations was tested by Tajima’s and D value, and the results were not significant, which were consistent with neutral mutation. There were no differences in generations 10, 15, 16, and 17for measured phenotypic traits. In other generations, for annual egg production, with G5, G10, G15, G16, G17>G0 (p<0.05). For age at the first egg and age at sexual maturity, with G10, G15, G16, G17>G5>G0 (p<0.05).

**Conclusion:**

Combined with the results of *COI* gene DNA barcodes, *MHC B-G* gene, and phenotypic traits we can see that genetic diversity remained stable from generations 10 to 17 and the equimultiple random matching pedigrees conservation population conservation effect of Langshan chicken was effective as measured by these criteria.

## INTRODUCTION

The Black Langshan, which originated in China, is a dual purpose fowl whose reproductive qualities suggest it as a pure breed of domesticated poultry [1]. The preservation of closed populations may be complex and it is difficult to avoid loss of genes when breeding groups are small [2]. Thus, conservation programs should consider the loss of genes [3]. According to genetic equilibrium theory, because inbreeding increases with generations, increasing the generation interval and size of breeding populations are essential to the preservation of existing gene pools [4], and thus a random mating system can reduce drift [5,6]. Tracking the separation and recombination of a target gene by measuring it’s variation from generation to generation, and reducing it’s loss due to genetic drift have significance for better protection of local chicken breeds [7]. Here, we report monitoring conservation effect on a Chinese indigenous chicken breed using major histocompatibility complex (MHC) and DNA barcords.

## MATERIALS AND METHODS

### Langshan (N line) population

Langshan (N line) chickens are mainly distributed in Jiangdu and Yangzhou city, Jiangsu Province, and Shanghai. The N line was initially introduced to the East China Academy of Agricultural Sciences in 1951 with a second introduction in 1952. The objective was to reduce age at sexual maturity and egg weight while maintaining other production qualities [8]. To accelerate performance, Langshan females were mated to Australian black males, a population imported by the East China Academy of Agricultural Sciences from New Zealand in 1949. It was a dual purpose chicken, whose age at the first egg was about 180 days with an annual egg number of about 160, and egg weight of 60 g. The feathers, shank, toe, and beak were black. The initial cross was backcrossed for 2 generations into the N line and the population was then closed for 4 generations. In 1959 it was then transferred to the Poultry Institute of the Chinese Academy of Agricultural Sciences, where it has been maintained as part of the National Chicken Genetic Resources (NCGR) [9].

### Equimultiple random matching pedigrees conservation (ERMPC) flock

Introduced in 1975, the equimultiple random matching pedigrees conservation (ERMPC) Langshan flock has been a conserved population since 2000 via equimultiple random matching pedigrees, which consists of 60 sire families of 60 males and 300 females. Thus a male to female ratio of 1:5. The flock was reproduced by artifical insemination. Each family had the same-sex numbers with basically the same distribution. When a hen had no progeny, individuals from other hens were randomly assigned to the male. Full and half sibling mating were avoided. All generations and breeding were conducted in the same facility. Production performance records include body weight at 300 days of age, age at the first egg, age at sexual maturity, and annual egg production based on the 300 females and 60 males used for conservation. Seventeen generations of Langshan ERMPC flock has been established until now.

### Monitoring of the genetic structure

At 180 days of age, blood samples were obtained from the brachial vein of the 60 males and 300 females used to reproduce the ERMPC flocks. Blood samples were not initially retained in each generation, however, blood samples were collected every fifth generations. Three consecutive generations (G15, G16, G17) of blood samples were subsequently collected for the purpose of testing the current changes in productivity and genetic diversity among the three consecutive generations.

All samples were collected as whole blood in 70% ethanol at room temperature. Genomic DNA was prepared according to Sambrook [10] and stored at 4°C. Genomic DNA samples were quantified spectrophotometrically and diluted to 100 ng/μL. Twenty individual blood samples were randomly selected for the MHC B-G loci polymorphism of different generations by illumina platform re-sequencing.

Based on the Genbank *MHC B-G* gene sequence (Accession number: NW_003763992) and Cox l (Accession number: AP003322), primers were designed using the application Oligo 6.0 software. All polymerase chain reaction (PCR) products (including negative control) are delivered to the electrophoresis chamber for routine electrophoresis. The quality of the primer was determined by the banding: whether the primer worked, the size of the product band was correct, and whether the dimer was serious. The poor quality primers were redesigned. After screening, 19 pairs of *MHC B-G* gene and 1 pair cytochrome oxidase I (*COI*) gene were selected (Table 1) and products monitored for allelic frequencies across generations.

### *MHC* B-G locus polymorphism analysis

The *MHC B-G* locus polymorphism of different generations was analyzed by illumina platform re-sequencing method, including illumina platform sequencing, establishment of DNA library, Miseq sequencing, and statistical analysis. The DNA library was constructed using multiple PCR method, and the amplification size was 300 to 400 bp. Different samples were distinguished by different barcode primers, and high-throughput sequencing was performed on the amplicons. Sequencing was performed using the Illumina MiSeq sequencing platform, the sequencing pattern Paired-end, and the sequencing read length was 2×300 bp. Sequence data were obtainedt by removing the connector sequence using the Cutadapt software (DOI http://dx.doi.org/10.14806/ej.17.1.200). Sequence alignment software BWA (v0.7.12) was used to compare the *MHC B-G* sequence after cleavage with the reference sequence [14]. The Genesis Analysis Toolkit (GATK) software was used for mutation detection (http://www.broadinstitute.org/gatk/guide/topic?name=best-practices).

### Cytochrome oxidase I gene polymorphism analysis

PCRs of *COI* gene were performed on 100 ng of genomic DNA in a 50 μL mixture containing 1×reaction buffer, 1 unit of high fidelity Taq polymerase (Promega, Madison, WI, USA), 2.0 mmol/L of Mg^2+^, 300 pmol of each dNTP, and 2.5 pmol forward and reverse primers. The PCR conditions used were 300 s at 95°C, 30 cycles of 50 s at 94°C, 50 s annealing at 58°C to 60°C and 50 s extension at 72°C, and a final extension step of 300 s at 72°C in a Perkin-Elmer thermocycler 9600 (Perkin Elmer, Norwalk, CT, USA).

PCR products were ligated into pMD18-T vectors and transformed into TOP10 cells using the TOPO TA Cloning Kit (Invitrogen, Carlsbad, CA, USA). Transformed bacteria were plated onto Media Amp Blue LB agar (Invitrogen, USA) containing ampicillin, X-gal, and isopropyl-β-D-thiogalactopyranoside. Pick a single white colony for culture, and clones identified as positive were picked for bi-directional sequencing by Shanghai Biological Engineering Co., Ltd (Shanghai, China). Sequences from each clone were aligned with GenBank sequences (Accession number: AP003322), and analyzed using Clustal.

### Statistics

According to the allele genotypes of each sample, the number of alleles, allele frequency (Pi), the average observed heterogeneity (Ho), the average expected heterozygosity (He), and polymorphic information content (PIC) of *MHC B-G* gene were obtained. Average PIC and He for generations 0, 5, 10, 15, 16, and 17 in ERMPC were compared using SPSS 16.0. Differences were considered significant at p<0.05.

Clustal and blast software sequence comparisons were made of the *COI* gene single nucleotide polymorphism (SNP) loci of Red Jungle Fowl in GenBank (AP003322). *COI* gene polymorphism and haplotypes were analyzed using DNAsp 4.1, the same sequence as the same type haplotype, as well as the *COI* gene sequences using Tajima’s D value neutral inspection [11].

## RESULTS

### *MHC B-G* gene polymorphism analysis in 6 generation populations

The full length of *MHC B-G* gene of 120 individuals in generations 0, 5, 10, 15, 16 and 17 was measured using re-sequencing. There were 292 SNPs identified in the six generations. There were 2 alleles on each SNP. Ho, He, and PIC of *MHC B-G* gene are shown in Table 2. Ho of *MHC B-G* gene in generations 15, 16, and 17 remained stable. There were differences in different generations for Ho of *MHC B-G* gene, with generations 15, 16, and 17>10>5>0. He and PIC of *MHC B-G* gene in generations 10, 15, 16, and 17 remained stable. The results showed that Ho in generations 10, 15, 16, and 17 of Langshan population were significantly higher than He. There were differences in different generations for He and PIC of *MHC B-G* gene, with generations 10, 15, 16, and 17 >5>0.

### Cytochrome oxidase I gene polymorphism in 6 generation populations

The total length of the mitochondrial *COI* gene of 190 individuals in generations 0, 5, 10, 15, 16, and 17 was measured. The PCR product length of 1,902 bp, the expansion of the target band was clear, no non-specific band and primer dimer, could be recovered for purification. The cloned and sequenced sequences were cloned and sequenced, and the full length sequence of *COI* was 1,551 bp. The sequence of *COI* was compared with that of red jungle chicken on GenBank.

**Figure 1:**
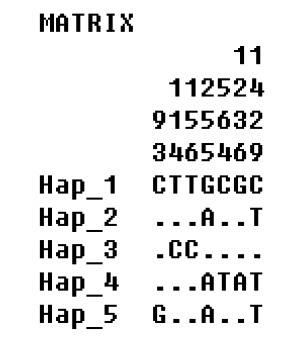
Variable sites in cytochrome oxidase I gene of haplotype in generations 0, 5, 10, 15, 16, 17 of Langshan conservation population. −, indicate the same base.

**Figure 2:**
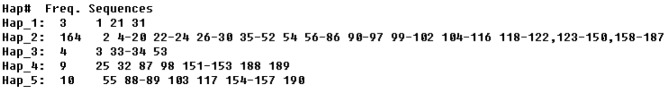
Haplotype frequency sequences of cytochrome oxidase I gene in generations 0, 5, 10, 15, 16, 17 of Langshan conservation population.

The sequence of *COI* The haplotype diversity, mean nucleotide differences and nucleotide polymorphisms of had the same 6 mutations in generations 0 and 5, and there were the same three mutations in generations 10, 15, 16, and 17. The haplotype distribution frequency is shown in Figure 1. It can be seen from Figure 2 and Table 3 that there were 5 haplotypes in generations 0, 5, 10, 15, 16, and 17, where Hap2 and Hap4 were the shared haplotypes, 164 individuals shared Hap2. Hap1 and Hap3 were the shared haplotypes in generations 0 and 5, with Hap5 shared haplotype in generations 10, 15, 16, and 17.

The haplotype diversity, mean nucleotide differences and nucleotide polymorphisms of *COI* sequences in different generations are shown in Table 4. The haplotypes diversity of *COI* gene in generations 0, 5, 10, 15, 16, and 17 were 0.276, 0.333, 0.119, 0.252, 0.269, and 0.312, respectively, and the average nucleotide differences (K) were 0.707, 1.083, 0.182, 0.314, 0.321, and 0.331. The nucleotide diversity of the *COI* gene was 0.00046, 0.00070, 0.00012, 0.00020, 0.00025, 0.00025, and 0.000284, respectively. The haplotype diversity, average nucleotide differences (K), nucleotide diversity (Pi) of 5th generation were highest, and there were no differences for generations 15, 16, and 17. There was higher genetic diversity in generation 5 and the generation 10 had less genetic diversity. The sequence of *COI* in the 6 generations tested by Tajima’s and D value were not significant, which is consistent for neutral mutation.

### Phenotypic traits analysis in 6 generations populations

There were no differences in generations 10, 15, 16, and 17 for measured phenotypic traits including body weight at 300 days old, age at the first egg, age at sexual maturity, and annual egg production (Table 5). In other generations, for annual egg production, with G5, G10, G15, G16, G17>G0 (p<0.05). For age at the first egg and age at sexual maturity, with G10, G15, G16, G17 >G5>G0 (p<0.05). There was no difference for boddy weight between generations 0 and 5.

## DISCUSSION

There is a considerable global interest in the conservation of indigenous poultry populations. In recent years, domestic and foreign scholars mainly used neutral molecular markers (microsatellites) to measure genetic diversity and monitor conservation effects [12,13]. They also found that the mitochondrial *COI* gene was relatively econserved with a certain variation. Thus, *COI* gene as DNA barcode in the identification of animal species had a certain feasibility and effectiveness [14,15], for the analysis of genetic diversity of a population [16,17]. Other studies have shown the correlation does not only select neutral and adaptive genetic variation [18,19]. Neutral genetic markers can not accurately reflect the adaptive genetic basis of the species, thus the evolutionary potential of the species studied from the point of view of adaptive genetic variation has become a cutting edge of conservation genetics.

The *MHC* not only has a role in animal immunity and disease resistance, but it can be a tool in population and conservation genetics [20,21]. This is because *MHC* adaptive genetic markers show a high degree of genetic diversity in the choice of equilibrium, and can reflect levels of genome variation [22,23].

Heterozygosity, called genetic diversity, reflects genetic variation on tested loci among populations, and is generally considered to be an appropriate parameter to measure genetic variation of population. Low heterozygosity reflects high genetic uniformity, and thus less genetic diversity. Ho in generations 10, 15, 16, and 17 of the Langshan population were significantly higher than He. Under normal circumstances, a certain degree of cross-amplification between the loci will lead to false He, making Ho greater than He [24].

With *MHC B-G* loci used in this study, the mean heterozygosity across all generations was ranged from 0.27 to 0.41. The highest was in generations 10, 15, 16, and 17, and the lowest (0.27) was in the 0 generation. The results of the heterozygosity were consistent with that of PIC. The lowest genetic diversity of the 0 generation maybe because the Langshan population had a high degree of inbreeding. The Langshan flock was established in 2000 with full and half sibling mating were avoided. He and PIC was increasing from 5th to 17th generation. The increase of genetic polymorphism of *MHC B-G* gene has created conditions for *MHC* gene to adapt to changing antigen. *MHC* gene polymorphism is an important molecular basis for its encoded protein involved in antigen presentation, and it plays a key role in adapting to the changing external environment [25,26]. The *MHC* genes are an essential component of the adaptive immune system, responsible for the recognition and presentation of foreign antigens [27]. He and PIC remained stable in generations 10, 15, 16, and 17. This may be because Langshan conserved population gradually adapted to the local environment, with better pathogen resistance, as reflected generations 10, 15, 16, and 17.

The variation of genetic diversity of *MHC B-G* gene in different generations of Langshan population was in accordance with the hypothesis of the choice of fluctuation (choice of spatiotemporal transformation). The fluctuation of species of the pathogen changes maintains the diversity of *MHC* in the population [28]. With the passage of time and spatial transformation, the species and diversity of pathogens will change accordingly, and thus host susceptibility to pathogenic bacteria and parasites will also change [29]. *MHC* polymorphisms can lead to population adaptations to endemic pathogenic micro-organisms if there are limited genetic exchanges or barriers to spatial communication between the populations. The Langshan conservation group has been conserved in the National Chicken Genetic Resources (NCGR). Thus, with the passage of time, the conservation population gradually adapted to the local pathogenic microorganisms. The genetic diversity of the *MHC B-G* gene of conservation population remained stable.

The full-length sequence of *COI* in Langshan conserved population was 98% to 100% similar to that of White Leghorn, Java Red chicken, Chinese Red Jungle and Burma Red Jungle. About 86% of individuals in generations 0 to 17 of Langshang chicken shared haplotype Hap2. For generations 10 to 17 sharing of Hap5 indicated that the *COI* barcodes were more stable in different generations. The sequence of *COI* in 6 generations was tested by Tajima’s D value, and not significant, which were consistent for a neutral mutation. Suggesting that there was no population expansion in the transmission process, and the population size was stable, which showed that the program was effective.

The lowest measured phenotypic traits including body weight at 300 days of age, age at the first egg, age at sexual maturity, and annual egg production in the 0 generation maybe because the Langshan population had a high degree of inbreeding and lower production performance. After 0 generation, with full and half sibling mating were avoided, the production performance of the 5th generation was gradually increased. The measured phenotypic traits were consistent across generations 10, 15, 16, and 17, which showed that Langshan conserved population had adapted to the local environment.

The results of *COI* gene DNA barcodes, *MHC B-G* gene, and measured phenotypic traits showed that genetic diversity remained stable from generation 10 to 17 and the ERMPC population conservation was effective as measured by these criteria.

At present, any method of detecting genetic diversity has its own advantages and limitations in theory or in practical research, and there is not a technique that can completely replace other methods [30]. Therefore, many molecular markers of neutral and adaptive mutation used to detect the genetic diversity will be able to better monitor the effect of conservation.

## Figures and Tables

**Table 1:** Primers for sequencing of the chicken *MHC B-G* gene (NW_003763992)

Primers	Forward primer	Reverse primer	Length of PCR product
B-G-1	CGGACCCTCTAAGAACGAT	CACGGAGAGATGGTGAGAT	370
B-G-2	TCCATGCCCCACATTAAC	TTCACCACAGCTTCTGCA	355
B-G-3	TGGCTCATACAGCTGTGC	GACAAGGGTTCCTTTTCCA	379
B-G-4	CCTTCGGGTTCTGTGATCT	CTATCTGCCCACCCTTACC	367
B-G-5	GGATTCCCAGTGCTCATT	GGAACCGATTGTGAAAGG	382
B-G-6	GGGATGTGTCAATCCTGG	CTTCCAATGGGAAGACCC	363
B-G-7	GGAGAAAGCTGCAGCATT	GATAGCGCTGCTTGTTCC	367
B-G-8	TCTCTGACCATGCACTGC	GCAATGCATGATGAGTGG	363
B-G-9	CTGTGTGAGCTGTGGGATC	GATGGTTGATGAGAGGAACAT	383
B-G-10	TCCAGAGAAAGACAGTGAAGAG	TCACCCATCTCTTCAGAGTG	365
B-G-11	CACTGCGTGTTGCTTTTC	CGCATGATGAGAGGAACAT	350
B-G-12	AGAGCGACTAGCTGCCAA	TTCTGAAAGTGTCTCTCTGGAA	350
B-G-13	TCTGACCATGCACTGCTT	GGAAAGCAGCACATGATG	366
B-G-14	AGACTTTGTGAGCTGTGGG	AGTGCACGATGAGAGGAAC	379
B-G-15	GTTGGGGTCTTCCTGTGA	GAGGTGGCACAGCTTGTC	376
B-G-16	TTCTTCCCTGTCCCAAAG	GAGGTGGCACAGCTTGTC	384
B-G-17	CCCGTCCCAAAGGACTAT	AGCTTGTCATCCCATGGA	374
B-G-18	GGTGAGTCTTTGTCCCCA	GAGTTGGAGGTCGCACAC	395
B-G-19	CTGCAGTTCTGTCAGCCA	ATGCATGGTGAGAAGGGT	389
COI	CCTAACGCTTCAACACTC	TTCAACTTCTTGGGCATC	1,902

MHC, major histocompatibility complex; PCR, polymerase chain reaction; COI, cytochrome oxidase I.

**Table 2:** Means and standard deviations for Ho, He, and PIC of *MHC B-G* genes in different generations

Genetic diversity	Generation					

	0	5	10	15	16	17
Average observed Ho	0.10±0.13^d^	0.29±0.23^c^	0.46±0.25^b^	0.53±0.24^a^	0.50±0.24^a^	0.53±0.30^a^
Average expected He	0.27±0.14^c^	0.36±0.12^b^	0.41±0.10^a^	0.43±0.08^a^	0.41±0.09^a^	0.41±0.11^a^
PIC	0.23±0.10^c^	0.29±0.08^b^	0.32±0.06^a^	0.33±0.05^a^	0.32±0.06^a^	0.32±0.07^a^

Ho, heterogeneity; He, heterozygosity; PIC, Polymorphic information content; *MHC B-G*, major histocompatibility complex B-G.

a–d Means in a row with different superscripts differ significantly (p<0.05).

**Table 3:** The number of haplotypes in different generations for the Langshan conservation population

Haplotype	Generation					

	0	5	10	15	16	17
Hap1	2	1	-	-	-	-
Hap2	23	22	31	30	28	30
Hap3	1	3	-	-	-	-
Hap4	1	1	1	1	3	2
Hap5	-	-	1	4	4	1

**Table 4:** The haplotype diversity, K and Pi of *COI* gene for the Langshan conservation population

Generation	No. of individuals	No. of haplotypes	Haplotype diversity	Average number of nucleotide differences (K)	The nucleotide diversity (Pi)	Tajima’s test D value (p value)
0	27	4	0.276	0.707	0.00046	−1.6005 (0.10>p>0.05)
5	27	4	0.333	1.083	0.00070	−0.8925 (p>0.10)
10	33	3	0.119	0.182	0.00012	−1.7282 (0.10>p>0.05)
15	35	3	0.252	0.314	0.00020	−1.2666 (p>0.10)
16	35	3	0.269	0.321	0.00025	−1.2761 (p>0.10)
17	33	3	0.312	0.331	0.00028	−1.2675 (p>0.10)

*COI*, cytochrome oxidase I.

**Table 5:** Means and standard deviation for phenotypic traits by generation

Traits	Generation					

	0	5	10	15	16	17
Body weight at 300 d	1,831.20±79.04^b^	1,900.40±144.27^ab^	1,972.20±155.12^a^	1,987.40±187.25^a^	1,995.70±227.03^a^	2,000.20±131.17^a^
Age at the first egg	163.97±6.73^c^	169.87±7.26^b^	175.17±4.72^a^	176.47±4.75^a^	176.65±8.30^a^	180.23±5.70^a^
age at sexual maturity	114.87±2.97^c^	116.87±3.00^b^	119.48±1.81^a^	119.87±1.63^a^	119.65±2.06^a^	120.13±3.20^a^
Annual egg production	142.67±10.76^b^	153.48±9.53^a^	156.07±8.94^a^	159.47±8.64^a^	158.45±8.21^a^	160.13±6.55^a^

a–c Means in a row with different superscripts differ significantly (p<0.05).
